# Anti-Obesity Effects of *Lactobacillus fermentum* CQPC05 Isolated from Sichuan Pickle in High-Fat Diet-Induced Obese Mice through PPAR-α Signaling Pathway

**DOI:** 10.3390/microorganisms7070194

**Published:** 2019-07-07

**Authors:** Kai Zhu, Fang Tan, Jianfei Mu, Ruokun Yi, Xianrong Zhou, Xin Zhao

**Affiliations:** 1 Chongqing Collaborative Innovation Center for Functional Food, Chongqing University of Education, Chongqing 400067, China; 2 Children’s Research Institute, Chongqing University of Education, Chongqing 400067, China; 3 Chongqing Engineering Research Center of Functional Food, Chongqing University of Education, Chongqing 400067, China; 4 Chongqing Engineering Laboratory for Research and Development of Functional Food, Chongqing University of Education, Chongqing 400067, China; 5 Department of Public Health, Our Lady of Fatima University, Valenzuela 838, Philippines; 6 College of Food Science, Southwest University, Chongqing 400715, China

**Keywords:** sichuan pickle, anti-obesity effect, PPAR-α signaling pathway, mice, expression

## Abstract

Sichuan pickle is a traditional fermented food in China which is produced by the spontaneous fermentation of Chinese cabbage. In this study, the anti-obesity effects of a new lactic acid bacterium (*Lactobacillus fermentum* CQPC05, LF-CQPC05) isolated from Sichuan pickles were assessed in vivo. An obese animal model was established in mice by inducing obesity with high-fat diet. Both serum and tissues were collected from the mice, and then subjected to qPCR and Western blot analyses. The results showed that LF-CQPC05 could decrease the values of hepatosomatic, epididymal fat, and perirenal fat indices that were induced by a high-fat diet in mice. Moreover, LF-CQPC05 reduced the levels of alanine aminotransferase (ALT), aspartate aminotransaminase (AST), total cholesterol (TC), triglyceride (TG), and low-density lipoprotein cholesterol (LDL-C), and increased the level of high-density lipoprotein cholesterol (HDL-C) in both serum samples and liver tissues of obese mice fed with a high-fat diet. Pathological observations demonstrated that LF-CQPC05 could alleviate the obesity-induced pathological changes in the liver tissue of mice, and reduce the degree of adipocyte enlargement. The results of qPCR and Western blot analyses further indicated that LF-CQPC05 upregulated the mRNA and protein expression levels of lipoprotein lipase (LPL), PPAR-α: peroxisome proliferator-activated receptor-alpha (PPAR-α), (cholesterol 7 alpha-hydroxylase) CYP7A1, and carnitine palmitoyltransferase 1 (CPT1A), and downregulated the expression levels of peroxisome proliferator-activated receptor-gamma (PPAR-γ) and CCAAT enhancer-binding protein alpha (C/EBP-α) in both liver tissue and epididymal adipose tissue. Taken altogether, this study reveals that LF-CQPC05 can effectively inhibit high-fat diet-induced obesity. Its anti-obesity effect is comparable to that of l-carnitine, and is superior to that of *Lactobacillus delbrueckii* subsp. *bulgaricus*, a common strain used in the dairy industry. Therefore, LF-CQPC05 is a high-quality microbial strain with probiotic potential.

## 1. Introduction

Traditional Sichuan pickle is produced mainly through lactic acid fermentation, along with acetic acid fermentation and ethanol fermentation, by soaking the vegetables with a specific concentration of salt water. Typically, the fermentation process is carried out in a high concentration of salt (5–10%) by beneficial microorganisms (mainly lactic acid bacteria) that are naturally attached to the surface of vegetables. The growth of other harmful microorganisms can be inhibited by the increased acid production, decreased pH value, and high osmotic pressure of salt [1]. In salt water, lactic acid bacteria can grow and produce acidic substances by utilizing sugar and nitrogen-containing substances, and also generate flavor components to enhance the flavors of pickles. A large number of lactic acid bacteria have been identified in pickles, which play a key role in regulating the flavor and quality of pickles [2]. Previous studies have shown that lactic acid bacteria isolated from fermented foods, such as pickles, exhibit a variety of health benefits, such as preventing constipation, colitis, liver damage, and diabetes, and can thus be considered as probiotics [3,4]. The functions of *Lactobacillus* that have been identified, for example, include the antioxidant role of *Lactobacillus fermentum* and *Lactobacillus coryniformis* [5], and the anti-constipation role of *Lactobacillus plantarum* YS4 in mice [6].

Lactic acid bacteria are the main fermentation microorganisms existing in traditional fermented foods, such as fermented dairy products, which can produce flavor substances including low-mass organic molecules of aldehydes, acids, and esters. Lactic acid bacteria promote the formation of flavor compounds in traditional fermented foods by fermenting carbohydrates, and are thus important for fermented foods [7]. Lactic acid bacteria are widely distributed in nature, and their treatment effects on intestinal diseases have been demonstrated by several studies, such as improved food digestibility and utilization rate, reduced serum cholesterol, control of endotoxin production, and inhibition of the growth and reproduction of spoilage bacteria and their products in intestinal tract [8,9]. Other studies have confirmed that lactic acid bacteria play crucial roles in regulating the physiological functions of the human body, such as maintaining gut microecological balance, boosting nutrient levels and promoting tissue development. Decreased probiotic bacteria and increased harmful bacteria may trigger the body’s responses, such as immune suppression and inflammatory cell accumulation, thus leading to a series of metabolic disorders [10]. Diet and other environmental factors can not only directly regulate body weight and insulin resistance, but also affect the composition of intestinal microbiota. High-fat diet could lead to a difference in the microbiota in mice. Among them, the representation of the phyla Firmicutes, Proteobacteria, and Verrucomicrobia increased significantly, while Bacteroidetes decreased significantly [11]. Bacterial imbalance also occurs in obese patients. The reduced microorganisms are mainly butyrate-producing bacteria and bacteria with sulfate metabolism and antioxidant activity [12]. At the same time, microbiota can induce the host to express genes related to lipid and carbohydrate metabolism, resulting in increased energy intake from the diet, and ultimately lead to host weight gain [13]. In addition to the direct impact on host metabolism, lactic acid bacteria also affect the absorption of nutrients in the intestinal tract, while nutrients also affect the intestinal microbiota. Intestinal microbiota is also an integral part of the host’s nutritional environment; intestinal microbiota can help improve the metabolic potential of the host and, in some cases, even serve as the host’s nutrition, thus producing beneficial effects on the host and forming a mutually reinforcing dependence with the host [14]. Considering these specific physiological activities, lactic acid bacteria have been widely used in food and medical industries.

Obesity is an important determinant that affect the health status of human beings, and has become a global health problem. Obesity is caused by the imbalance of food intake and energy consumption. The occurrence of obesity is associated with genetic factors, endocrine disorders, metabolic abnormalities, and nutritional imbalance. Obesity is difficult to study due to the great variation between subjects. Finding out the gene pathways associated with obesity and targeting genes to lose weight through represent both a difficulty and a hotspot of research [15]. The excessive intake of high-sugar and high-fat ingredients in modern convenience foods can lead to overnutrition, which further aggravates obesity. In addition, obesity has been linked to other metabolic diseases, including type 2 diabetes and cardiovascular and cerebrovascular diseases [16]. Therefore, controlling lipid metabolism and preventing obesity can help to reduce the risk of metabolic diseases [17]. As a chronic metabolic disease, obesity is caused by an imbalance between energy intake from the diet and energy expenditure [18]. The high-fat diet induction of C57BL/6J mice is a classic model of obesity, which simulates the unhealthy dietary habits of human beings, in which the fat accumulation, increased serum total cholesterol (TC) and triglyceride (TG) levels, and insulin resistance result from the intake of high-fat and high-calorie foods, together with less exercise [19]. Some studies have shown that in vivo administration of PPAR-γ agonist substances can stimulate adipocyte differentiation and fatty acid oxidation, promote weight loss, and reduce adipocytes and adipose tissue, thus achieving an anti-obesity effect [20,21]. Further studies have reported that the PPAR-α pathway is associated with free fatty acid-induced lipid accumulation in hepatocytes, and upregulated expression of fatty acid oxidation-related genes (e.g., PPAR-α and CPT1); while SREBP-1 downregulation can decrease free fatty acid-induced lipid accumulation in hepatocytes, regulate lipid metabolism, and inhibit weight gain [22]. Besides, probiotic strains or fermented dairy products have been shown to alter intestinal microbiota composition, attenuate intestinal inflammation, and decrease intestinal permeability, thereby affecting organ function and reducing the serum levels of cholesterol, visceral fat, and triglycerides, leading to an excellent lipid-lowering effect [23,24,25,26].

In this study, a *Lactobacillus fermentum* strain was isolated and identified from Sichuan pickles, which was named *Lactobacillus fermentum* CQPC05 (LF-CQPC05). The lipid-lowering effect of this *Lactobacillus fermentum* strain on high-fat diet-induced obese mice was investigated by determining the serum and tissue indices. l-carnitine can promote the conversion of fat into energy. Taking l-carnitine can reduce body fat and weight without reducing water and muscle. It is a kind of non-side-effect fat-reducing nutritional health product. In this study, it was used as a positive control to evaluate the effect of LF-CQPC05. In addition, the mechanisms underlying the protective effect of LF-CQPC05 on the lipid metabolism pathway in obese mice were further elucidated. The findings would help clarify the anti-obesity mechanism of LF-CQPC05, and provide a theoretical basis for further in-depth clinical research and industrial development.

## 2. Materials and Methods

### 2.1. Experimental Strain

In this study, *Lactobacillus fermentum* CQPC05 (LF-CQPC05) was isolated from Sichuan pickles sold in Chongqing, China. Its identity was confirmed using BLAST (Basic Local Alignment Search Tool) in NCBI and stored in China General Microbiological Culture Collection Center (Beijing, China), the preservation number of LF-CQPC05 was CGMCC No. 14494. *Lactobacillus delbrueckii* ssp. *bulgaricus* (preservation number AB200048) was purchased from China Center for Type Culture Collection (Wuhan, Hubei) and was used as a positive control strain for comparison. The resistance of lactic acid bacteria to artificial gastric juice and bile salt were determined using previously published in vitro experiments [9].

### 2.2. Establishment of Animal Model

Fifty 6-week-old SPF C57/BL6J mice (male/female ratio = 1:1) were obtained from the Experimental Animal Center of Chongqing Medical University (SCXK (Yu) 2017-0001). After 1 week of adaptation, the mice randomly were divided into 5 groups (10 mice in each group; male/female ratio = 1:1): normal, model, *Lactobacillus fermentum* CQPC05 (LF-CQPC05), l-carnitine (positive control drug), and *Lactobacillus delbrueckii* subsp. *bulgaricus* (LDSB). The mice in the normal group were fed a low-fat diet (10% fat mixed diet, Changzhou Qihang Biotechnology Co., Ltd., Changzhou, Jiangsu, China, Table 1), while the mice in the other four groups were fed a high-fat diet (45% fat mixed diet, Table 1) for 8 weeks. In addition, the mice in LF-CQPC05 and LDSB groups were intragastrically administered with 1.0 × 10^9^ CFU/kg of LF-CQPC05 and LDSB, respectively, daily for 8 weeks. Meanwhile, the mice in l-carnitine group were intragastrically administered with 200 mg/kg (b·w) of l-carnitine daily for 8 weeks daily. The body weights of mice were recorded weekly during the entire experiment. After 8 weeks, all mice were fasted for 24 hours, then the blood samples were collected from the heart, and the liver and epididymal fat cells were isolated for subsequent analyses. Moreover, the weights of liver tissue, epididymal adipose tissue, and perirenal adipose tissue were measured, and the organ indices were calculated according to the following equation: organ index = organ mass (g)/mouse body mass (kg). This study was conducted in accordance with the Declaration of Helsinki, and the protocol was approved by the Ethics Committee of Chongqing Collaborative Innovation Center for Functional Food (201804003B) on 19 April 2018 (Chongqing, China).

### 2.3. Determination of Alanine Aminotransferase (ALT), Aspartate Aminotransaminase (AST), Total Cholesterol (TC), Triglyceride (TG), High-Density Lipoprotein Cholesterol (HDL-C), and Low-Density Lipoprotein Cholesterol (LDL-C) Levels in Serum and Liver Samples

After centrifuging at 4000 rpm for 10 min at 4 °C, the serum in the upper phase was separated and collected. The serum levels of alanine aminotransferase (ALT), aspartate aminotransaminase (AST), TC, TG, high-density lipoprotein cholesterol (HDL-C) and low-density lipoprotein cholesterol (LDL-C) were determined using commercial kits (Nanjing Jiancheng Bioengineering Institute, Nanjing, Jiangsu, China) according to the manufacturer’s instructions. The 10% liver homogenates were centrifuged at 4000 rpm for 10 min at 4 °C, and the isolated supernatant was used for the determination of liver tissue index according to the instructions of the used kits (Nanjing Jiancheng Bioengineering Institute, Nanjing, China).

### 2.4. Pathological Observation of Liver Tissue and Epididymal Adipose Tissue

The liver and epididymal adipose tissues of mice were isolated and fixed in 10% formalin solution for 48 h. The tissues were dehydrated, cleared, waxed, embedded, sliced and stained with hematoxylin and eosin (H&E). The morphological differences of the tissues were observed under an optical microscope (BX43; Olympus, Tokyo, Japan).

### 2.5. Quantitative PCR (qPCR) Assay

The liver tissue of mice was milled using TissueRuptor, and TRIzol^TM^ Reagent (Thermo Fisher Scientific, Inc., Waltham, MA, USA) was used to extract total RNA from the tissue samples. Total RNA was diluted to a final concentration of 1 μg/μL, and cDNA synthesis was performed using 1 μL of the diluted total RNA samples. Subsequently, 1 μL of cDNA was mixed with 10 μL of SYBR Green PCR Master Mix (Thermo Fisher Scientific, Inc., Waltham, MA, USA), 1 μL each of upstream and downstream primers (Table 2), and 7 μL of distilled water. The used primers were designed using primer information from NCBI (National Center for Biotechnology Information, Bethesda, MD, USA) and produced by Thermo Fisher Scientific [27,28]. After mixing, the qPCR reaction was conducted as follows: 95 °C for 60 s; 40 cycles of 95 °C for 15 s, 55 °C for 30 s, and 72 °C for 35 s; and a final step of 95 °C for 30 s and 55 °C for 35 s. glyceraldehyde-3-phosphate dehydrogenase (GAPDH) was used as the internal reference gene, and 2^−ΔΔCt^ method was used to calculate the relative expression of the target genes [29].

### 2.6. Western Blot Analysis

Liver tissue (100 mg) and 1 mL of RIPA buffer were homogenized using TissueRuptor at 12,000 r/min, for 5 min at 4 °C, and then centrifuged at 12,000 rpm/min for 15 min at 4 °C. The intermediate phase protein was isolated, and the total protein concentration was determined using the BCA method. The samples from each group were diluted to 50 μg/mL, and then the diluted protein was mixed with sample buffer at 4:1 and heated for 5 min at 100 °C. The stacking gel and separation gel for SDS-PAGE were prepared by mixing acrylamide, resolving buffer, stacking buffer, distilled water, 10% APS, and TEMED, and then cast for gel electrophoresis. Prestained protein ladder and the samples were added into different wells of the gel, and then separated for 50 min using a vertical gel electrophoresis system. The PVDF membrane was activated by methanol for 1 min prior to blotting. The PVDF membrane was blocked with 5% skim milk in 1× TBST solution for 1 h. After blocking, the PVDF membrane was washed with 1× TBST (Thermo Fisher Scientific, Inc., Waltham, MA, USA), and then incubated with the primary antibody at 25 °C for 2 h. Then, the PVDF membrane was washed 5 times with 1× TBST, and incubated with the secondary antibody at 25 °C for 1 h. Finally, the PVDF membrane was incubated with SuperSignal West Pico PLUS chemiluminescent substrate, and then visualized using Tanon Luminescent Imaging Workstation (Shanghai Tanon Technology Co., Ltd., Shanghai, China) [30].

### 2.7. Statistical Analysis

Measurement of the serum and tissue samples was conducted three times in parallel, and the mean value was then calculated. Statistical analysis was performed using the SAS version 9.1 statistical software package (SAS Institute Inc., Cary, NC, USA). To verify normal distribution and homogeneity of values, Shapiro–Wilk and Levene’s tests were used. Group means were checked by the analysis of variance followed using Tukey’s honestly significant difference. *p* values of 0.05 were considered statistically significant.

## 3. Results

### 3.1. Resistance of Lactic Acid Bacteria to Artificial Gastric Juice and Bile Salt

Table 3 showed that the survival rate in artificial gastric juice at pH 3.0 and 0.3% bile salt of LF-CQPC05 were much higher than those of LDSB. LF-CQPC05 had good anti-artificial gastric acid and bile salt effect in vitro.

### 3.2. Body Weight of Mice

**Figure 1:**
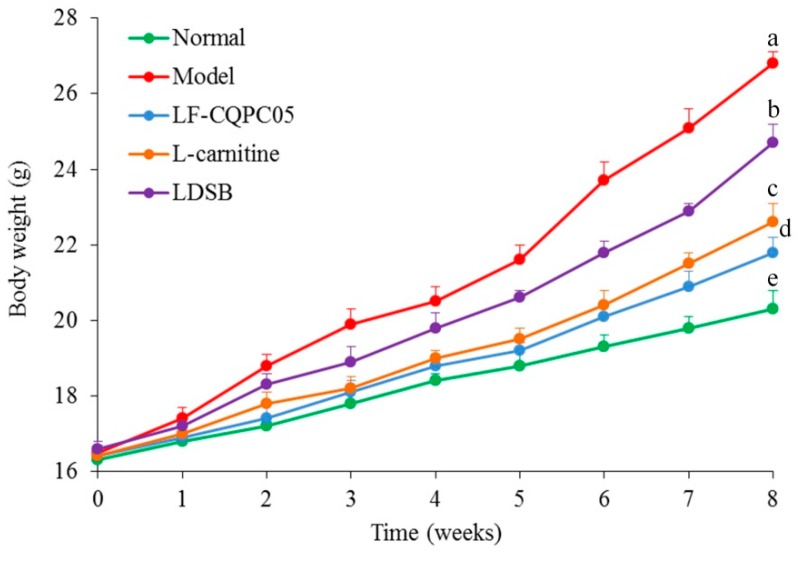
Effect of LF-CQPC05 on body weight gain in mice. Values presented are the mean ± standard deviation (N = 10/group). Sample data in each group come from a normal distribution, the difference in variance between the two groups was significant (*p* < 0.05). ^a–e^ Mean values with different letters are significantly different (*p* < 0.05) according to Tukey’s honestly significant difference. LDSB: Mice treated with *Lactobacillus delbrueckii* subsp. *bulgaricus* (1.0 × 10^9^ CFU/kg); LF-CQPC05: Mice treated with *Lactobacillus fermentum* CQPC05 (1.0 × 10^9^ CFU/kg); l-carnitine: l-carnitine (200 mg/kg).

Table 4 showed that there was no significant difference in dietary intake between groups during the experiment and the differences in mouse body weight were not caused by different dietary intake. As shown in Figure 1, the mice fed a low-fat diet (normal group) maintained a normal body weight. The weight gain of mice fed a high-fat diet (model group) was increased compared to the normal group. Besides, the body weights of mice in l-carnitine, LF-CQPC05, and LDSB groups were lower than those in model group. In addition, the body weight of mice in LF-CQPC05 group was decreased compared to l-carnitine group and LDSB group. The results indicate that LF-CQPC05 can reduce the body weight of mice under high-fat diet conditions, and thus exerts an inhibitory effect on obesity.

### 3.3. Organ Indices of Mice

Table 5 showed that the hepatosomatic, epididymal adipose, and perirenal adipose indices of the model group were significantly higher (*p* < 0.05) than those of normal group, indicating that high-fat diet may result in the enlarged liver and the increased amount of epididymal and perirenal adipose tissues. Moreover, the liver, epididymal fat, and perirenal fat indices of the LF-CQPC05, l-carnitine, and LDSB groups were significantly lower (*p* < 0.05) compared to the model group. The lipid lowering activities of LF-CQPC05 and l-carnitine were remarkably effective, which were comparable to the low-fat and normal diet groups. Therefore, LF-CQPC05 can effectively suppress the increase in organ index caused by high-fat diet induced obesity, and alleviate the enlargement of body tissues caused by high-fat contents.

### 3.4. Levels of ALT, AST, TC, TG, HDL-C, and LDL-C in the Serum of Mice

As shown in Table 6, the serum levels of ALT, AST, TC, TG, and LDL-C were decreased, and the HDL-C level was increased in the normal group, while the opposite trends were found in the model group. After LDSB, LF-CQPC05, and l-carnitine treatments, the levels of ALT, AST, TC, TG, and LDL-C were significantly decreased (*p* < 0.05), and the level of HDL-C was significantly increased (*p* < 0.05) in obese mice. Notably, the levels of ALT, AST, TC, TG, HDL-C, and LDL-C in LF-CQPC05 and l-carnitine groups were similar to those in the normal group. These findings reveal that LF-CQPC05 can attenuate high-fat diet-induced obesity in mice, and the effect was similar to that of l-carnitine and even better than that of LDSB.

### 3.5. Pathological Observation of Liver and Epididymal Adipose Tissue of Mice

**Figure 2:**
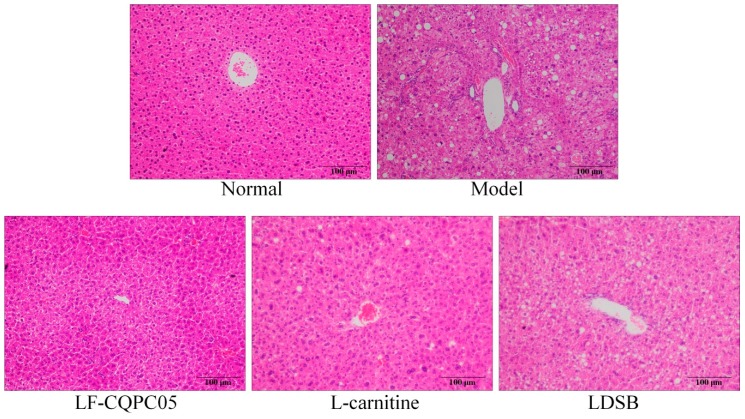
H&E pathological observation of hepatic tissue in mice. Magnification 100×. LDSB: Mice treated with *Lactobacillus delbrueckii* subsp. *bulgaricus* (1.0 × 10^9^ CFU/kg); LF-CQPC05: Mice treated with *Lactobacillus fermentum* CQPC05 (1.0 × 10^9^ CFU/kg); l-carnitine: l-carnitine (200 mg/kg).

After H&E staining, there was no abnormal changes in hepatic cells, such as steatosis, of the normal group, and the structure of liver tissue was clear and intact, the structure of hepatic lobule was normal, the cell boundaries were clear, and the nucleus was located in the cell center (Figure 2). Meanwhile, in the model group, the liver tissue exhibited microvesicular steatosis and high fat content, fat vacuoles were observed around blood vessels, cell swelling was triggered, and cell wall integrity was damaged. After treatment with LF-CQPC05, the liver tissue steatosis in mice was alleviated as compared to the model group, and the fat vacuoles within hepatocytes were fewer and smaller, and the cell morphology was relatively similar to that of the normal group.

**Figure 3:**
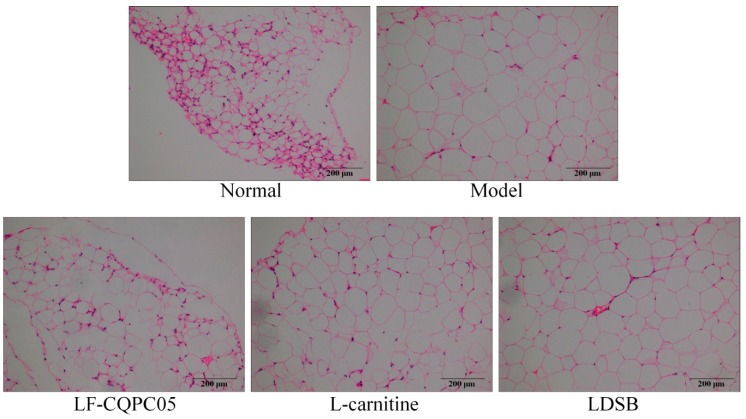
H&E pathological observation of epididymal tissue in mice. Magnification 100×. LDSB: Mice treated with *Lactobacillus delbrueckii* subsp. *bulgaricus* (1.0 × 10^9^ CFU/kg); LF-CQPC05: Mice treated with *Lactobacillus fermentum* CQPC05 (1.0 × 10^9^ CFU/kg); l-carnitine: l-carnitine (200 mg/kg).

The staining results of epididymal adipose tissue are shown in Figure 3. Notably, the adipocytes of the normal group were smaller and neatly arranged, while the adipocytes of the model group were larger and their cell membranes were thinner. The adipose tissues of mice treated with LF-CQPC05 and l-carnitine were denser compared to the model group, and the average size of adipocytes was similar to that of the normal group, indicating that the anti-obesity effect of LF-CQPC05 is superior to that of LDSB treatment. Taken together, these results suggest that LF-CQPC05 could reduce the fat cell hypertrophy caused by a high-fat diet.

### 3.6. Expression of RNA and Protein in Mouse Liver Tissue

**Figure 4:**
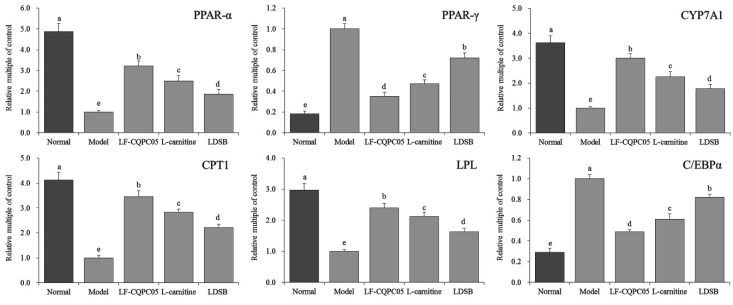
PPAR-α, PPAR-γ, CYP7A1, CPT1, LPL, and C/EBPα mRNA expression in hepatic tissue of mice. Sample data in each group come from a normal distribution, and the difference in variance between the two groups was significant (*p* < 0.05). ^a–e^ Mean values with different letters in the bar are significantly different (*p* < 0.05) according to Tukey’s honestly significant difference. LDSB: Mice treated with *Lactobacillus delbrueckii* subsp. *bulgaricus* (1.0 × 10^9^ CFU/kg); LF-CQPC05: Mice treated with *Lactobacillus fermentum* CQPC05 (1.0 × 10^9^ CFU/kg); l-carnitine: l-carnitine (200 mg/kg).

**Figure 5:**
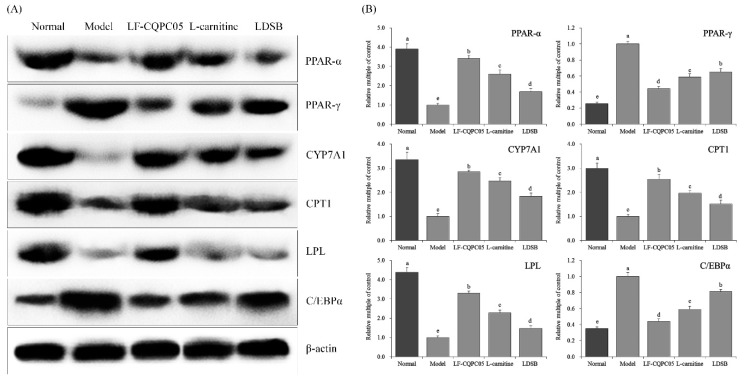
The PPAR-α, PPAR-γ, CYP7A1, CPT1, LPL, and C/EBPα protein expression in hepatic tissue of mice. (**A**) Protein expression; (**B**) Relative quantification of band. Sample data in each group come from a normal distribution, the difference in variance between the two groups was significant (*p* < 0.05). ^a–e^ Mean values with different letters in the bar are significantly different (*p* < 0.05) according to Tukey’s honestly significant difference. LDSB: Mice treated with *Lactobacillus delbrueckii* subsp. *bulgaricus* (1.0 × 10^9^ CFU/kg); LF-CQPC05: Mice treated with *Lactobacillus fermentum* CQPC05 (1.0 × 10^9^ CFU/kg); l-carnitine: l-carnitine (200 mg/kg).

As shown in Figure 4 and Figure 5, the mRNA levels of LPL, PPAR-α, CYP7A1, and CPT1 were downregulated, and the expression levels of PPAR-γ and C/EBP-α were upregulated in the liver tissue of model group. On the contrary, the expression levels of these genes in the normal group showed the opposite trends, in which the expression levels of LPL, PPAR-α, CYP7A1, and CPT1 were increased, while the expression levels of PPAR-γ and C/EBP-α were decreased. The treatment of LDSB, LF-CQPC05, and l-carnitine significantly reduced the expression levels of LPL, PPAR-α, CYP7A1, and CPT1, and significantly increased the expression levels of PPAR-γ and C/EBP-α (*p* < 0.05) in the liver tissue of obese mice. It was noted that the effects of LF-CQPC05 and l-carnitine were better compared to LDSB. Taken together, LF-CQPC05 can ameliorate high-fat diet-induced obesity in mice by regulating both mRNA and protein levels, and the effect is relatively similar to that of l-carnitine, which is greater than the commonly used commercial strain LDSB.

## 4. Discussion

Since this study aimed to simulate the effect of LF-CQPC05 on adults, we chose half of the males and half of the females to carry out the experiment. LSDB is the most commonly used lactic acid bacteria in dairy products. In order to compare the efficacy of LF-CQPC05 with that of this common strain, LSDB was selected as the positive control strain in this study. For weight loss experiments, animal body mass is one of the most intuitive indices. Additionally, the organ indices can also reflect the obesity and intestinal lipid metabolism to a certain extent. Moreover, weighing the epididymal white adipose tissue and calculating the fat/body mass ratio can reflect the degree of obesity in mice [31]. After obesity induction in mice, the weight of white fat and the proportion of white fat in body weight are increased. High-fat diet can cause a stress response in the body, resulting in liver lipid accumulation and, ultimately, hepatomegaly and deteriorated liver function. Under normal conditions, the synthesis and excretion of lipids in hepatocytes maintain a dynamic balance, with no lipid accumulation and lipid droplet formation. However, when lipid accumulates in the cytoplasm, lipid droplets of varying sizes can be formed, which will devastate the normal structure of hepatocytes and thus affect liver function. Therefore, the measurement of mouse organ indices can directly reflect changes in the structure or function of organs, which allows for the evaluation of anti-obesity agents on established obese mice models. The results of this study showed that high-fat diet significantly increased the organ indices of mice, while LF-CQPC05 and l-carnitine effectively alleviated such effects and further decreased the weight gain of obese mice, which were comparable to those of normal mice.

Liver is an important organ for detoxification and lipid metabolism. Serum levels of ALT and AST are important determinants of liver function, and these enzyme markers can indicate the abnormal degree of liver injury [32]. ALT and AST are mainly distributed in hepatocytes. During hepatocyte necrosis, ALT and AST are released into the blood circulation, which increases the level of serum enzymes and coincides with the degree of hepatocyte injury, resulting in their indices being the most effective, at present, for measuring liver function [33]. Given that blood lipid content can reflect the state of systemic lipid metabolism, abnormal blood lipids (e.g., TG, TC, HDL-C, and LDL-C) are one of the most common markers of metabolic syndrome [34]. Excessive accumulation of body fat is the main factor contributing to obesity. Approximately 30–50% of individuals with high body fat are associated with fatty liver, which is usually comprised of reversible lesions that can be improved by early diagnosis and treatment [35]. In this study, it was found that the contents of ALT, AST, TG, TC, and LDL-c had increased significantly in the mice fed with a high-fat and high calorie diet, leading to severe hepatic vacuolation and lipid deposition and, ultimately, obesity. Treatment with LF-CQPC05 could decrease the hepatic levels of ALT, AST, TG, TC, and LDL-C, as well as attenuate lipid deposition and increase HDL-c content in obese mice.

PPAR is a member of the nuclear receptor superfamily that regulates the expression of target genes, and can be activated by ligands such as fatty acid-like compounds. PPAR plays important roles during cell signal transduction processes, and is involved in a wide variety of metabolic pathways such as oxidative respiration and fatty acid β-oxidation, as well as the physiological and pathological processes of various diseases, including obesity [36]. There are three types of PPARs: α, β, and γ. PPAR-γ is highly expressed in adipose tissues, and regulates the processes of adipocyte proliferation and differentiation. PPAR-γ not only affects the expression of other lipid metabolism-related genes, but is also a main regulator for adipocyte gene expression and insulin signal transduction [37]. A synergistic effect was found between PPAR-α and PPAR-γ, and the activation of PPAR-γ can induce the expression of PPAR-α. Moreover, C/EBP-α is a transcription factor that plays an essential role in regulating the differentiation of adipocytes. Its expression is positively correlated with the expression of PPAR-γ [38]. In this study, LF-CQPC05 could regulate the expression of PPAR-α, PPAR-γ, and C/EBP-α in obese mice, which were close to that of normal mice. It could be seen that LF-CQPC05 could cause weight reduction through regulating the expression of PPAR-α, PPAR-γ, and C/EBP-α.

CPT1 is a crucial rate-limiting enzyme in the process of fatty acid oxidation (FAO). The change in CPT1 level is closely related to the development of obesity. The C-terminal of CPT1 is located on the outer membrane of mitochondria, whereas the N-terminal is on the cytoplasm side. CPT1 controls the rate of FAO by converting acyl coenzyme A to acyl carnitine, and CPT2 converts acyl carnitine to acyl coenzyme A during FAO [39]. Interestingly, PPAR-α is the upstream transcription factor in FAO, and CPT-1 is its key downstream target gene. The expression of CPT-1 in liver is regulated by its upstream factor PPAR-α, and the hepatic lipid metabolism pathway PPAR-α/CPT-1 is an important pathway associated with obesity. PPAR-α can accelerate the transport of fatty acids to mitochondria by inducing the expression of CPT1 in both muscle and liver, and ultimately promote the β-oxidation of fatty acids. PPAR-α is also involved in the β-oxidation and ω-oxidation of mitochondria by regulating the expression levels of acetyl coenzyme A oxidase and cytochrome P450. Thus, the PPAR-α/CPT-1 pathway plays a pivotal role in the regulation of lipid metabolism in mitochondria, and thereby inhibits the development of obesity [40]. Therefore, LF-CQPC05 could also regulate the expression of CPT1 and reduce the body weight of obese mice, thus playing a role in weight loss.

LPL is a proteolytic enzyme that serves as the key enzyme in lipid metabolism. The major functions of LPL are to catalyze the decomposition of chylomicrons (CM) and very-low-density lipoproteins (VLDL) to TG and free fatty acids, and to promote the transport of protein, phospholipid, and apolipoprotein. As a consequence, the level of HDL is increased, and the high level of TG is suppressed in obesity [41]. A previous study has shown that a decrease in LPL activity may result in the reduced decomposition of TG-rich VLDL and CM, slow clearance rate, increased plasma TG levels, and inhibited HDL-C formation, thus leading to hypertriglyceridemia, low HDL-C level and, ultimately, obesity [42]. Therefore, LF-CQPC05 could also affect the expression of LPL, thus regulating the weight of mice and inhibiting weight gain.

CYP7A1 is a rate-limiting enzyme for the synthesis of bile acids and catalyzes the decomposition of cholesterol into bile acids in the liver [43]. This enzyme belongs to the liver-specific microsomal P450 superfamily. More than half of the cholesterol in human body is converted into bile acid by CYP7A1 and then excreted from the body. Therefore, CYP7A1 gene is the most important regulatory gene in cholesterol synthesis pathway and is involved in maintaining cholesterol homeostasis, synthesizing bile acids, and preventing obesity [44]. Therefore, LF-CQPC05 can upregulate the expression of CYP7A1 in obese mice, thereby regulating the weight loss of mice. In the present study, LF-CQPC05 upregulated the mRNA and protein expression levels of LPL, PPAR-α, CYP7A1, and CPT1, and downregulated the expression levels of PPAR-γ and C/EBP- in the liver of obese mice, and thus alleviated high-fat diet-induced obesity by reducing fat accumulation in the mouse body.

PPAR-α can regulate fatty acid metabolism in the liver. The PPAR-α-based pathway is an important pathway for controlling fat metabolism. In this study, *Lactobacillus fermentum* CQPC05 is better at regulating the PPAR-α pathway than general lactic acid bacteria (LDSB), so it may play a better role in reducing fat.

There are a large number of intestinal microorganisms in the human intestinal tract. Those that are beneficial can regulate the metabolism of sugar and lipid and their metabolites by themselves [45]. A clinical study has also shown that dietary intervention of intestinal microbiota can make obese patients lose weight, and improve blood lipid and other indicators [46]. Further studies have also shown that food-borne microorganisms can enhance immunity and reduce weight by interfering with intestinal microbiota [47,48]. Food-borne microorganisms in this study also have similar effects and have the value of scientific research and utilization.

Poor dietary habits, high-fat and low-fiber diet, and excessive intake of food additives can destroy the microecological health of the intestinal tract and cause intestinal inflammation. Long-term high-fat diet will lead to the increase of harmful bacteria in the intestine, decrease of beneficial bacteria, destruction of intestinal health, obesity, and other diseases. Dietary supplements, including yogurt, fermented foods, and healthy foods (purslane, *Myrciaria dubia*, etc.) can increase intestinal probiotics to a certain extent and protect the intestinal tract. The direct supplementation of probiotics is a more direct and better way to protect intestinal health and reduce weight [48,49,50]. In this study, LF-CQPC05 isolated from fermented food was used as food-borne probiotics to supplement and reduce weight in mice.

Intestinal microbiota is one of the most important determinants of metabolic disorders such as obesity. Obesity-causing diets can completely change the bacterial population, leading to metabolic disorders. Rebuilding intestinal microbiota and improving intestinal health can eliminate metabolic disorders caused by obesity and play a role in weight loss. Probiotics can have beneficial effects in preventing and treating obesity and related metabolic disorders by directly affecting the mucosal barrier and surrounding cells [49]. At the same time, supplementation of probiotics to improve intestinal microbiota can play a role in regulating obesity-related inflammation, thereby playing a role in weight loss as a probiotic [48]. In addition, good intestinal microbiota can also increase energy consumption to prevent visceral and liver fat deposition, thus maintaining body weight and keeping the body healthy. In the case of imbalance of intestinal microbiota, this effect can be achieved by supplementing probiotics or promoting functional food to improve intestinal microbiota [50]. In this study, LF-CQPC05 could also clear fat in the liver and inhibit obesity-related inflammation. It might also play a role in weight loss through antioxidant and other effects. Further research on the mechanism of LF-CQPC05 will be needed to further strengthen these findings.

## 5. Conclusions

In this study, the anti-obesity effect of LF-CQPC05 in mice was investigated. Through the establishment of an obese mouse model by high-fat diet, the protective effects of LF-CQPC05 on the obesity-stimulated imbalance of serum and liver tissue indices was evaluated. The findings revealed that LF-CQPC05 could effectively improve the abnormal lipid metabolism in serum and liver tissues, and attenuate obesity-induced liver damage. The results of animal experiments showed that LF-CQPC05 could significantly control high-fat diet-induced obesity in mice, thereby decreasing the risk of lipid metabolic disorders. Therefore, it can be concluded that LF-CQPC05 is a microbial resource with excellent biological activity in mice, and which can interfere with obesity by regulating lipid metabolism and thus achieving its liver protection and anti-obesity effects. The data of this study lays the foundation for further studies on LF-CQPC05, but mainly limited to animal experimentation. Hence, the anti-obesity effect of LF-CQPC05 should be further confirmed with human clinical trials.

## Figures and Tables

**Table 1:** Diet formula of mice in this study.

Diet Ingredients	10% Fat Mixed Diet (%)	45% Fat Mixed Diet (%)
Corn flour	24.89	7.79
Bran	15.00	7.00
Wheatmeal	7.00	
Soybean meal	18.50	22.00
Sucrose	16.38	20.00
Lard	1.02	19.50
Premix	17.21	23.71

Premix: trace mineral elements, vitamins, synthetic amino acids.

**Table 2:** Sequences of primers used in this study.

Gene Name	Sequence
PPAR-α	Forward: 5′-CCTCAGGGTACCACTACGGAGT-3′
	Reverse: 5′-GCCGAATAGTTCGCCGAA-3′
PPAR-γ	Forward: 5′-AGGCCGAGAAGGAGAAGCTGTTG-3′
	Reverse: 5′-TGGCCACCTCTTTGCTGTGCTC-3′
CYP7A1	Forward: 5′-AGCAACTAAACAACCTGCCAGTACTA-3′
	Reverse: 5′-GTCCGGATATTCAAGGATGCA-3′
CPT1	Forward: 5′-AAAGATCAATCGGACCCTAGACA-3′
	Reverse: 5′-CAGCGAGTAGCGCATAGTCA-3′
LPL	Forward: 5′-AGGGCTCTGCCTGAGTTGTA-3′
	Reverse: 5′-AGAAATCTCGAAGGCCTGGT-3′
C/EBPα	Forward: 5′-TGGACAAGAACAGCAACGAGTAC-3′
	Reverse: 5′-GCAGTTGCCCATGGCCTTGAC-3′
GAPDH	Forward: 5′-ACCCAGAAGACTGTGGATGG-3′
	Reverse: 5′-ACACATTGGGGGTAGGAACA-3′

PPAR-α: peroxisome proliferator-activated receptor-alpha; PPAR-γ: peroxisome proliferator-activated receptor-gamma; CYP7A1: cholesterol 7 alpha-hydroxylase; CPT1: carnitine palmitoyltransferase 1; LPL: lipoprotein lipase; C/EBPα: CCAAT enhancer-binding protein alpha; GAPDH: glyceraldehyde-3-phosphate dehydrogenase. Primers were designed using the primer information of NCBI.

**Table 3:** Resistance of lactic acid bacteria to artificial gastric juice and bile salt.

Treatment	Survival Rate in Artificial Gastric Juice at pH 3.0 (%)	Survival Rate in 0.3% Bile Salt (%)
LF-CQPC05	83.22 ± 3.61	13.37 ± 1.17
LDSB	41.33 ± 2.74	8.65 ± 0.56

LDSB: Lactobacillus delbrueckii subsp. bulgaricus; LF-CQPC05: Lactobacillus fermentum CQPC05.

**Table 4:** Food intake of mice during the experiment (g/day).

Group	1^st^ Week	2^nd^ Week	3^rd^ Week	4^th^ Week	5^th^ Week	6^th^ Week	7^th^ Week	8^th^ Week
Normal	3.25 ± 0.11 ^a^	3.32 ± 0.14 ^a^	3.62 ± 0.13 ^a^	3.92 ± 0.15 ^a^	4.12 ± 0.19 ^a^	4.33 ± 0.22 ^a^	4.55 ± 0.26 ^a^	4.88 ± 0.27 ^a^
Model	3.29 ± 0.08 ^a^	3.33 ± 0.16 ^a^	3.59 ± 0.12 ^a^	3.89 ± 0.18 ^a^	4.15 ± 0.22 ^a^	4.36 ± 0.24 ^a^	4.61 ± 0.28 ^a^	4.92 ± 0.23 ^a^
LF-CQPC05	3.22 ± 0.10 ^a^	3.35 ± 0.15 ^a^	3.63 ± 0.17 ^a^	3.95 ± 0.15 ^a^	4.21 ± 0.16 ^a^	3.29 ± 0.23 ^a^	4.59 ± 0.18 ^a^	4.93 ± 0.25 ^a^
l-carnitine	3.26 ± 0.12 ^a^	3.32 ± 0.14 ^a^	3.66 ± 0.16 ^a^	3.93 ± 0.16 ^a^	4.23 ± 0.23 ^a^	3.35 ± 0.25 ^a^	4.66 ± 0.19 ^a^	5.02 ± 0.27 ^a^
LDSB	3.31 ± 0.14 ^a^	3.33 ± 0.17 ^a^	3.61 ± 0.19 ^a^	3.94 ± 0.16 ^a^	4.19 ± 0.20 ^a^	3.32 ± 0.24 ^a^	4.60 ± 0.25 ^a^	5.05 ± 0.22 ^a^

Values presented are the mean ± standard deviation (N = 10/group). Sample data in each group come from a normal distribution, and the difference in variance between the two groups was significant (*p* < 0.05). ^a^ Mean values over the same column are not significantly different (*p* < 0.05) according to Tukey’s honestly significant difference. LDSB: Mice treated with *Lactobacillus delbrueckii* subsp. *bulgaricus* (1.0 × 10^9^ CFU/kg); LF-CQPC05: Mice treated with *Lactobacillus fermentum* CQPC05 (1.0 × 10^9^ CFU/kg); l-carnitine: l-carnitine (200 mg/kg).

**Table 5:** Organ index of mice in each group (N = 10).

Group	Hepatosomatic Index	Epididymal Adipose Index	Perirenal Adipose Index
Normal	32.38 ± 0.71 ^e^	10.30 ± 0.53 ^e^	1.56 ± 0.17 ^e^
Model	48.32 ± 1.20 ^a^	28.16 ± 2.08 ^a^	12.13 ± 1.26 ^a^
LF-CQPC05	35.36 ± 1.03 ^d^	14.25 ± 1.81 ^d^	3.41 ± 0.48 ^d^
l-carnitine	38.23 ± 1.12 ^c^	18.33 ± 1.65 ^c^	5.20 ± 0.62 ^c^
LDSB	42.39 ± 1.06 ^c^	23.11 ± 1.76 ^b^	8.39 ± 0.54 ^b^

Values presented are the mean ± standard deviation (N = 10/group). Sample data in each group come from a normal distribution, the difference in variance between the two groups was significant (*p* < 0.05). ^a–^^e^ Mean values with different letters over the same column are significantly different (*p* < 0.05) according to Tukey’s honestly significant difference. LDSB: Mice treated with *Lactobacillus delbrueckii* subsp. *bulgaricus* (1.0 × 10^9^ CFU/kg); LF-CQPC05: Mice treated with *Lactobacillus fermentum* CQPC05 (1.0 × 10^9^ CFU/kg); l-carnitine: l-carnitine (200 mg/kg).

**Table 6:** Levels of alanine aminotransferase (ALT), aspartate aminotransaminase (AST), total cholesterol (TC), triglyceride (TG), high-density lipoprotein cholesterol (HDL-C), and low-density lipoprotein cholesterol (LDL-C) in serum of mouse (N = 10).

Group	ALT (U/L)	AST (U/L)	HDL-C (mmol/L)	LDL-C (mmol/L)	TC (mmol/L)	TG (mmol/L)
Normal	17.08 ± 1.33 ^e^	11.02 ± 0.89 ^e^	1.08 ± 0.12 ^a^	0.48 ± 0.06 ^e^	1.62 ± 0.21 ^e^	0.47 ± 0.04 ^e^
Model	60.35 ± 3.87 ^a^	51.88 ± 2.13 ^a^	0.28 ± 0.06 ^e^	1.97 ± 0.32 ^a^	5.86 ± 0.52 ^a^	1.70 ± 0.11 ^a^
LF-CQPC05	29.82 ± 1.86 ^d^	18.01 ± 1.12 ^d^	0.83 ± 0.06 ^b^	0.71 ± 0.08 ^d^	2.21 ± 0.24 ^d^	0.68 ± 0.08 ^d^
l-carnitine	35.17 ± 1.38 ^c^	25.62 ± 1.53 ^c^	0.62 ± 0.05 ^c^	0.93 ± 0.07 ^c^	3.03 ± 0.25 ^c^	0.93 ± 0.10 ^c^
LDSB	48.71 ± 2.02 ^b^	34.19 ± 1.66 ^b^	0.49 ± 0.05 ^d^	1.34 ± 0.16 ^b^	4.18 ± 0.22 ^b^	1.36 ± 0.12 ^b^

Values presented are the mean ± standard deviation (N = 10/group). Sample data in each group come from a normal distribution, the difference in variance between the two groups was significant (*p* < 0.05). ^a–^^e^ Mean values with different letters over the same column are significantly different (*p* < 0.05) according to Tukey’s honestly significant difference. LDSB: Mice treated with *Lactobacillus delbrueckii* subsp. *bulgaricus* (1.0 × 10^9^ CFU/kg); LF-CQPC05: Mice treated with *Lactobacillus fermentum* CQPC05 (1.0 × 10^9^ CFU/kg); l-carnitine: l-carnitine (200 mg/kg).
